# Potato (*Solanum tuberosum* L.) cultivars physiological, biochemical performance and yield parameters response to acid mine water irrigation and soil physiochemical properties

**DOI:** 10.1038/s41598-024-52507-4

**Published:** 2024-01-23

**Authors:** Rabelani Munyai, David M. Modise

**Affiliations:** 1 Department of Agriculture and Animal Health, College of Agriculture and Environmental Sciences, Horticulture Research Centre, University of South Africa, Private Bag X6, Florida, 1710 South Africa; 2 School of Agricultural Sciences, Faculty of Natural and Agricultural Sciences, North-West University, Private Bag X6001, Potchefstroom Campus, Potchefstroom, 2520 South Africa

**Keywords:** Plant sciences, Environmental sciences

## Abstract

This paper aimed to analyse the potato cultivar’s response to physiological, biochemical performance, yield parameters and soil physiochemical properties when subjected to quicklime (un)treated acid mine drainage (AMD) irrigation. A randomized design experiment was conducted with five water treatment levels: TW1; TW2; TW3; TW4 to TW5 replicated four times. The results showed that the quicklime treatment increased the pH of the AMD water, reduced the concentration of EC, NO_3_^−^, SO_4_^2−^ and ameliorated heavy metals. However, unsafe levels of heavy metals above the maximum permissible (WHO/FAO) levels were found in Pb, Mg and Mo for water (TW4 and TW5), while As, Cd and Cr for soils (ST4 and ST5) respectively. For potato tubers (TT4 and TT5) concentrations of As, Cd, Cr, and Pb were above the maximum levels. Stomatal conductance, chlorophyll content and yield parameters responded positively by increasing significantly on TW4 and TW5 treatments, but negatively (reduced) towards TW2 and TW3 treatments. A higher bioaccumulation factor was obtained for Zn ˃ Cu ˃ Mg ˃ Pb ˃ Mn, which was an indication of the contamination status of soil, with Zn being more concentrated than other metals. The findings indicate that quicklime-treated AMD is usable for potato irrigation with regular monitoring of heavy metal levels and strict observation of water reuse protocols. The use of this large source of ameliorated (AMD) water will go a long way in improving food security in South Africa and/or in countries where agriculture production is around mining areas.

## Introduction

With increased levels of water scarcity and its supply variability, the ability of the world to meet the growing demand for food for more people with fewer available resources per capita has become a major policy concern^1^. The scarcity has led to the need to consider the utilisation of alternative water sources including those discharged from industrial, commercial, and domestic activities. Interestingly, the practice has been reported to increase in recent years, particularly in countries where access to or availability of freshwater is limited. The utilization of wastewater not only conserves freshwater resources for domestic purposes such as drinking water and irrigation, but it also reduces pollution in adjacent bodies of water and the environment^2–5^. South Africa is ranked among 30 of the driest countries in the world and is expected to experience severe water scarcity in the future. To increase the sources of water, there are several proposals for alternatives including the re-use of treated wastewater. The country hosts plenty of abandoned and operational mines that drain acid mine drainage (AMD) water mostly into proximal waterbodies^7–9^. Although mining is a major contributor to the country's GDP, its activities can result in the release of by-products that have negative impacts on the fauna and flora of environments that surround mines^10^. As a result, there is a critical need to reduce toxins linked to AMD by implementing appropriate technology, eliminating waste, and implementing reuse and recycling strategies. As a result, there is a critical need to reduce toxins linked to AMD by implementing appropriate technology, eliminating waste, and implementing reuse and recycling strategies. When treated, AMD water can potentially be used for multiple purposes including the irrigation of crops and serve as an innovative solution for the current and future water crisis^11^.

In South Africa, agriculture accounts for more than 60% of water utilisation for its irrigation practices^12^. However, several published studies with varying successes have reported the use of (un)treated AMD for agricultural purposes^9,13–21^. Although some of the results have shown that it can have positive effects, in the main, the majority of the literature revealed negative effects largely caused by the activity of heavy metals. In South Africa^20^, reported that potato tubers (*Solanum tuberosum* L.) of Fianna and Lady Rosetta cultivars accumulated unsafe levels of Ni, Zn, and Sr when irrigated with Fly ash-treated AMD water. A published study by^22^ recorded higher concentrations of Cr, Ni, Cu, As, Cd, and Pb in potato (*Solanum tuberosum*), red onion (*Allium cepa*), and wild carrot (*Daucus carota*) established in multi-metal-contaminated soils relative to that recommended by the FAO/WHO, an indication that consumption of such crops could pose a risk. The findings from ^23^ showed significantly higher concentrations of Cd and Pb in rice grain, vegetables, and soybeans compared to the maximum permissible level in the vicinity of the Dabaoshan mine, located in southern China. When irrigating with mine wastewater^24^, revealed that the grain of winter wheat had significantly higher Cr, Pb, Cu and Zn relative to their counterparts irrigated with tapwater, thus implying that the irrigation with mine wastewater could result in the accumulation of heavy metals in wheat grain. When plants are exposed to stressful environmental conditions, their physiological and biochemical performances are altered^25^. For instance, a study by ^26^ examined the effects of irrigating wheat with mine wastewater (leachate of coal gangue, coal-washing wastewater, and precipitated coal-washing wastewater) on soil enzymes, physiological properties, and potential risks of heavy metal contamination. The results showed that mine wastewater irrigation caused adverse effects on rhizospheric enzymes, physiological properties, and grain yield of the winter wheat. Similarly, when wheat was supplied with mine wastewater, its growth, grain yield, leaf area, dry mass per stem, root activity, and net photosynthetic rate were markedly decreased relative to that irrigated with tap water^24^. However, in another study^27^, reported a significant increase in the height, spike length, grains spike and grain yield of wheat grown with the application of quicklime.

Potatoes (*Solanum tuberosum* L.) along with rice (*Oryza saliva* L.) and wheat (*Triticum aestivum* L.), are a significant staple food in various parts of the world and require an adequate supply of water to achieve a high-quality yield^28^. One of the most critical factors affecting potato yield and quality is the supply or availability of good unpolluted soil water^29^. In their results^30^, found that the potato crop is highly vulnerable to water stress, particularly during the tuber formation and tuber bulking growth stages and these may decrease the yield. Overall, the foremost factor that negatively influences the production of potatoes is the type of irrigation^31^. As one of the major crops, the cultivated potato is consumed each day by millions of people and the quality of the potato, thus, affects human health greatly. Therefore, the transfer of toxic elements from soils to plants is of great concern. Therefore, heavy metal contamination in agricultural soils, their transfer in a soil-potato system and physiological response have been of increasing concern.

In the present study, two potato cultivars Marykies and Royal were used for experimental investigation and a 3:1:1 Culterra topsoil mixture was irrigated with quicklime-treated AMD water. There are limited scientific experimental reports on the evaluation of potential heavy metals on soil properties, physiological parameters, and biochemical performance on Marykies and Royal potato cultivars when subjected to quicklime-treated acid mine drainage irrigation under greenhouse conditions.

### Research objectives

This study analysed the potato cultivar's physiological, biochemical and yield parameters response and soil physicochemical properties when subjected to quicklime treatments of AMD irrigation.

### The relevance of the study

This study’s findings are timely because South Africa has a water shortage and needs to utilize AMD to irrigate food crops. According to studies ^32^, AMD treated with lime can be used as an alternate source of irrigation water for food crops. In essence, irrigation of food crops with AMD treated with quicklime can elicit varying physiological and metabolic responses as well as rhizospheric microbial richness and diversity. It is therefore the uniqueness of this combination that merits reporting. Research on the irrigation of potatoes with quicklime-treated AMD produces results that serve as a blueprint to guide the effects such an alternative technology has on the quality of potatoes. This study seeks to address the potential of quicklime-treated AMD water for the irrigation of commercial potato cultivars. It will enable making informed decisions related to the use of treated AMD water in crop growing practices under changing climatic conditions and water deficit seasons of the current times.

## Materials and methods

First, we confirm that all methods including experiments and analyses were performed in accordance with relevant protocols, guidelines, and regulations. These methods were approved by the University of South Africa, and we also confirm that informed and ethical consent was obtained from all relevant institutions.

### Experimental study area, water sampling, pre-treatment, and physicochemical analysis

The greenhouse experimental study was conducted at the University of South Africa (UNISA), Florida Science Campus, Johannesburg, Gauteng Province (S 26° 10′ 30″ S, 27° 55′ 22.8″ E) over two growing seasons, the first season from August to November 2018 and second season from February to May 2019. The greenhouse's temperature ranged from 20 to 25 degrees, which was aligned with the requirements for potato growth. Potato seeds were donated from McCain Delmas Mpumalanga, South Africa. Before planting, water samples were collected from a gold mine, Mogale City in Gauteng and accurately measured into 2 L (L) containers. A total of five experimental treatments were used, however, only two different solution ratios (amount (g) of quicklime (QL) and 100 percentage of AMD) as shown below:

1. Treatment 1 (TW1) = 0:0, Tapwater.
2. Treatment 2 (TW2) = 0:100, AMD water.
3. Treatment 3 (TW3) = 1:100, 1 g Quicklime and AMD water.
4. Treatment 4 (TW4) = 2:100, 2 g Quicklime and AMD water
5. Treatment 5 (TW5) = 2:75:100, 2 g Quicklime, 75% FA and AMD water.

**Figure 1:**
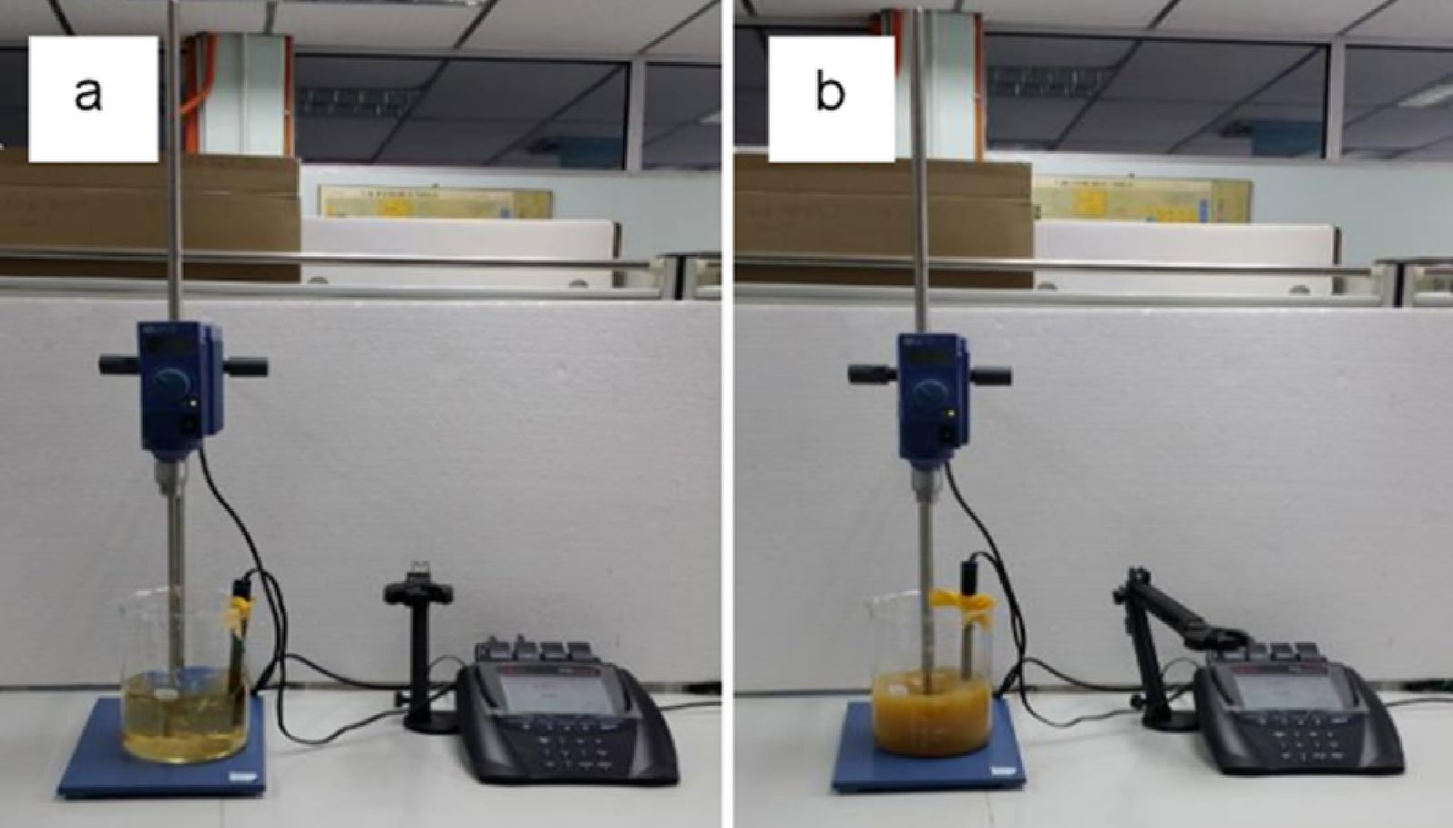
Jar test showing AMD water treatment used for irrigation (**a**) before and (**b**) after reaction between AMD and quicklime (Source: adapted from^33^).

Before irrigating with (un)treated AMD, as alluded to above, the water was treated with quicklime powder following^33^ protocol. Quicklime (QL) was obtained from All Lime Services Pty located at Elandsfontein in Johannesburg. For the Lab segment of the experiment, 2.5 g weight of QL was added into a 1 L beaker that contained 100% AMD water (1 g of QL equivalent to 1 L of AMD water). The AMD water that contained QL was stirred using a mechanical stirrer. The method to carry out the experiments is known as the Jar test, a well-known active treatment technique (Fig. 1). After the QL was added, the AMD water colour changed to orange and precipitation was simply observed. Triplicate water samples from each experimental treatment level were denoted as TW1 to TW5. The physicochemical characteristics which included pH, electric conductivity (EC), DO, NO_3_^–^, SO_4_^2–^ were recorded using a pH meter (A329, Thermo Scientific, Indonesia) and ICP-EOS (Agilent Technologies 700 series ICP-OES). The concentration of micronutrients (Cu, Fe, Mn, and Zn) and heavy metals (As, Cd, Co, Cr, Ni, Mg, Mn, and Mo) were analysed using an inductively coupled optical emission spectrometer (Agilent Technologies 700 series ICP-OES, USA).

### Experimental soil sampling and analyses

A mixture of 3:1:1 of Culterra topsoil, vermiculite and river sand was used for planting potato seeds. The soil samples were collected immediately before planting and after harvesting from soil layers of 0–20 cm. They were collected in triplicates from each treatment, air-dried through a 2 mm mesh sieve and preserved in plastic bags before analysis. A similar method as above was used after harvesting before the measurements of the physicochemical parameters.

### Pot experimental design, potato planting schedule and irrigation water treatments

**Figure 2:**
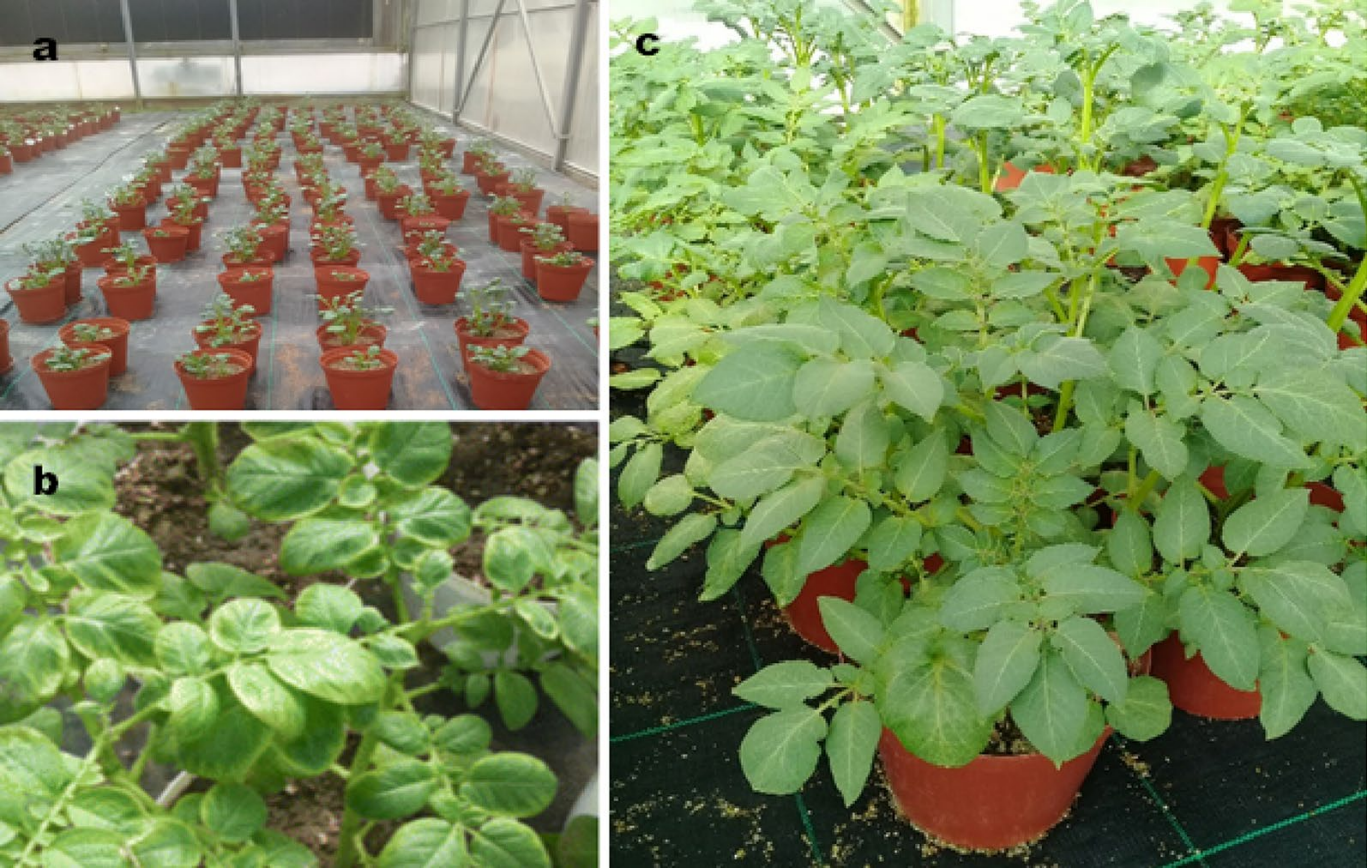
A randomized block design greenhouse (**a**) pot experimental setup for Royal and Marykies potato seeds, (**b** and **c**) symptoms of heavy metal effect shown by the initiation of discolouration in leaf margins in both cultivars. (Photo: Rabelani Munyai, 2018).

The Marykies and Royal potato cultivars were grown in the pots using a statistical approach suggested by^20^ in the greenhouses. The factorial experiment was randomized and designed into blocks which comprised six pots (2 × 5), in which potato tubers of almost equal diameters between 30 and 60 mm were planted in each 25 × 25 cm pot (Fig. 2a–c). After planting, from emergence until crop maturity, irrigation with the various AMD treatments (TW1–TW5) was applied every two days (until senescence). An Irrometer Soil Moisture Meter (SN: 946,776) (Model 30–KTCD–NL, Riverside, California) was used to accurately schedule irrigation. When the Irrometer reading was between 60 and 100 centibars, 500 mm irrigation water was applied to all experimental pots at every cycle.

### Physiological, biochemical and yield parameters measurements

To determine how irrigation with quicklime-treated AMD water affects the physiological and biochemical traits of the two potato varieties (Marykies and Royal), plant growth parameters, including yield, chlorophyll content, and stomatal conductance (DAP) were measured. The 4th matured completely expanded leaf (abaxial and adaxial) apex of the two potato cultivars was used for both chlorophyll content and stomatal conductance measurements at weekly intervals from 40 to 72 days after planting using a hand-held Minolta SPAD (Soil Plant Analysis Development: 502 m, 2900 PDL, Spectrum Technologies, Inc.) and Porometer (Model SC–1 Leaf Porometer, Pullman, USA) respectively. All tubers were rinsed with tap water after harvesting, dried with paper towels, and weighed. The UW4200H top loading Balance scale was used to determine the tuber fresh weights per plant. Fresh tubers per individual plant were freeze-dried and weighed to determine tuber dry matter using a freeze dryer (Free Zone Plus 2.5 L Cascade Benchtop Freeze Dry System Vacutec, USA).

### Determination of levels of heavy metals on potato tubers

To evaluate the effect of heavy metal content on the potato tuber, hydrochloric-nitric acids HNO_3_–HCl method adopted from^34^ was used. The method was employed because hydrochloric-nitric acids, HNO_3_–HCl, have shown to be the best acid combination suitable for potato tuber samples due to their capacity to liberate metal ions from such complex matrices of tuber materials and, as a result, to limit noise levels during the detection technique. A UW4200H top loading balance scale was used to weigh a total of 1 g potato tubers powder from each of the five treatments. The samples were then placed in microwave-safe jars and mixed with 9 mL of nitric acid (65%) and 3 mL of hydrochloric acid (37%). The digesting process, as previously mentioned by^35^, was carried out at 175 °C for 60 min at 6 W energy. The materials were digested, allowed to cool, and then centrifuged for 10 min at 10,000×g. The supernatant was then collected, brought up to a volume of 50 mL in each tube, and diluted with deionized water (1:3) before being filtered through the Whatman No. 1 filter paper. Overnight, the suspension settles at room temperature before the measurement of the concentration of micronutrients (Cu, Fe, Mn, and Zn) and heavy metals (As, Cd, Co, Cr, Ni, Mg, Mn, and Mo) using an inductively coupled optical emission spectrometer (Agilent Technologies 700 series ICP-OES, USA).

### Bioaccumulation of metals in soil-potato system

To understand the relationship of the bioaccumulation of metals in the soil-potato system, the soil-to-plant transfer can be predicted using a transfer factor (PTF)^36^. Metal concentrations in the extracts of soils and potatoes were calculated based on dry weight. The soil-to-plant transfer factor was calculated as^37^.

1 $${\varvec{TF}} = \frac{{{\mathbf{Cpotato}}\; {\mathbf{tuber}}}}{{{\mathbf{Csoil}}}}$$

where Cpotato tuber and Csoil represent the heavy metal concentration in extracts of plants and soils on a dry weight basis, respectively. The transfer coefficient may differ considerably between plant, soil, and metal types under investigation^38^.

### Statistical analysis

Data were subjected to two-way analysis of variance (ANOVA) carried out using TIBCO Statistica version 14.0 (StaSoft Inc., Tulsa, OK, USA) package (2020). Means separation was done using Tukey’s Honest Significant Difference (HSD) at p < 0.05.

## Results and discussion

### Physicochemical properties of the water and soil

Physicochemical parameters of different water treatments and soils recorded in the present study are summarised in Table 1 with a significant difference at p < 0.05 observed across the 5 treatments for all the measured parameters. After the application of quicklime in the AMD water, the pH value increased from 3.85 (TW2) to 6. 23–8.63 (TW3–TW4) and 8.85 (TW5), respectively. These values are within the permissible limit of World Health Organization (WHO) standards^39^. According to previous research^33,40–42^ quicklime and fly ash treatments have been shown to raise water pH to values suitable for crop irrigation. This is explained by the two substances' capacity to neutralize the acid produced by the AMD^43^. The EC and Total Dissolved Solids (TDS) values of treated water (TW3, TW4 and TW5) decreased when compared to untreated water (TW2); and were also within the recommended limit^39^. The study by^44^ reported that high EC values might make it difficult for plants to absorb ions from the soil solution. While, the TDS has a significant impact on plant growth, yield, and quality the values ranged from 853.14 to 2431.16 mg/L, and the EC of treated mine water ranged from 421.64 (TW4), 434.61 (TW5), to 917.43 (TW3). A study by^45^ showed a positive correlation between EC and TDS. This could account for the concurrent decrease of both parameters in this study under the ameliorating effect of QL and FA (Table 1). Only TW3 (1 g of QL) exceeded the maximum permitted limits for irrigation water’s EC (700 S/cm) and TDS (1000 mg/L). The investigation done by^9^ reported high EC (3100–13,000 S/cm) values of treated mine water that were over the maximum allowable limit for irrigation water in another investigation. Similar to the current study’s findings, the EC values of the examined samples were taken from mine wastewater at Eshidiya Mines in South Jordan and ranged from 3689 to 3795 S/cm with a mean value of 3724 12 S/cm, while those from another mine wastewater site ranged from 3869 to 3960 S/cm with a mean value of 3919 11 S/cm, both of which were above the WHO standard value^46^. This suggests that irrigation of crops with water that has been treated with 1 g of QL (TW3) is not recommended. The salinity appropriateness of irrigation water is assessed using the EC, or specific conductance, of a water sample. Increased soil salt levels caused by excessive soil salinization can harm crops^47^. Growing plants in salty conditions can restrict or hinder their ability to absorb water and nutrients, which results in stunted growth and lower yields. As a result of this study’s findings, it was determined that the selected potato cultivars could be watered with a solution made by combining AMD with 2 g of QL (TW4) and 2 g of QL and spiking with 75% of FA (TW5). Quicklime and fly ash treatment for AMD precipitates the sulphates linked to it, resulting in a decrease in its concentration^40,48^. In agreement with our findings, quicklime, and fly ash treatments (TW4 and TW5) reduced the sulfates compared to irrigation with raw AMD (TW2). Values, however, remained over the irrigation standard that is advised^39^. Sulfate can hinder a plant’s ability to absorb other nutrients when present in high concentrations, as reported by^49^. For the studied values, the physiochemical properties of soil irrigated with treated AMD water were significant (p < 0.05) among treatments (Table 1). In comparison to untreated AMD (TW2) and tapwater (TW1), there was a significant (p < 0.05) rise in the pH of the soil irrigated with treated AMD with ST3:5.67, ST4: 6.70, and ST5:7.23 respectively. In addition^50^, also noted a rise in pH value for soil irrigated with treated AMD water, which is consistent with our findings. Soil pH has a significant impact on the mobility and bioavailability of heavy metals^51^. The pH plays a significant role in plant health and growth by changing the chemistry of the soil, especially when it comes to boosting the number of nutrients that are available in the soil^52^.

**Table 1:** Physiochemical properties and heavy metals of quicklime (un)treated AMD irrigation water and irrigated soils.

Mean concentration of irrigation physiochemical properties of water (TW) levels					
Parameters	Treatments				
	TW1	TW2	TW3	TW4	TW5
pH	8.45 ± 0.11c	3.98 ± 0.01e	6.23 ± 0.06d	8.63 ± 0.06b	8.85 ± 0.08a
EC (µS/cm)	45.98 ± 0.98e	3641.33 ± 52.05a	917.43 ± 3.75b	421.64 ± 4.93d	434.61 ± 5.29c
TDS (mg/L)	128.35 ± 1.89e	4874.00 ± 24.27a	2431.16 ± 71.70b	922.61 ± 1.44c	846.47 ± 4.65d
NO_3_^−^ (mg/L)	2.17 ± 0.13e	6.29 ± 0.19a	2.34 ± 0.06d	2.38 ± 0.33c	2.66 ± 0.10b
DO (mg/L)	16.09 ± 0.19a	5.54 ± 0.18e	11.13 ± 0.29d	13.24 ± 0.40c	14.84 ± 0.21b
SO_4_^2−^ (mg/L)	224.55 ± 3.86e	5255.33 ± 49.08a	1127.55 ± 3.16d	1182.28 ± 14.62c	1195.81 ± 1.72b

Mean concentration of irrigated soil physiochemical properties (STW) levels					
Parameters	ST1	ST2	ST3	ST4	ST5
pH	7.13 ± 0.12a	3.85 ± 0.14d	5.67 ± 0.11c	7.70 ± 0.05b	7.87 ± 0.06a
EC (mS/m)	0.52 ± 0.09e	163.40 ± 2.77a	50.29 ± 1.49d	72.32 ± 1.47c	85.00 ± 0.95b
NO_3_^−^ (mg/kg)	0.74 ± 0.08e	8.78 ± 0.31a	4.21 ± 0.08b	2.80 ± 0.02c	2.17 ± 0.06d
SO_4_^2−^ (mg/kg)	16.25 ± 0.43e	12,706.01 ± 19.60a	848.32 ± 1.86d	1208.90 ± 16.92c	1264.99 ± 9.06b

Mean concentration of heavy metals on irrigation water (TW) levels					
Parameters	TW1	TW2	TW3	TW4	TW5
As	0.02 ± 0.00d	2.06 ± 0.11a	1.78 ± 0.14b	0.02 ± 0.00d	0.09 ± 0.01c
Cd	0.01 ± 0.00d	1.36 ± 0.02a	0.08 ± 0.00b	0.01 ± 0.00d	0.03 ± 0.00c
Co	0.03 ± 0.00c	6.57 ± 0.05a	2.40 ± 0.02b	0.03 ± 0.00c	0.02 ± 0.00d
Cr	0.04 ± 0.00c	3.78 ± 0.00a	0.66 ± 0.01b	0.04 ± 0.00c	0.03 ± 0.00d
Cu	0.06 ± 0.00d	1.72 ± 0.12a	0.11 ± 0.00c	0.01 ± 0.00e	0.14 ± 0.00b
Fe	3.73 ± 0.01d	1029.45 ± 13.87a	22.97 ± 0.29b	2.57 ± 0.03e	4.52 ± 0.01c
Mg	27.94 ± 0.99e	294.41 ± 3.39d	453.18 ± 3.07a	377.18 ± 1.66c	409.44 ± 1.67b
Mn	0.02 ± 0.00d	34.14 ± 0.34a	4.96 ± 0.05b	0.02 ± 0.00d	0.06 ± 0.00c
Mo	0.06 ± 0.01d	0.05 ± 0.00e	129.27 ± 1.01c	285.71 ± 1.73b	349.03 ± 1.74a
Ni	0.02 ± 0.00e	6.50 ± 0.35a	3.33 ± 0.03b	0.15 ± 0.01c	0.08 ± 0.00d
Pb	0.02 ± 0.00e	0.33 ± 0.01d	8.30 ± 0.30a	6.28 ± 0.01b	6.10 ± 0.01c
Zn	0.73 ± 0.01e	50.32 ± 0.66a	5.94 ± 0.08b	2.09 ± 0.05c	1.16 ± 0.05d

Mean ± SE in the same row with dissimilar letters are significantly different at p < 0.05.

DO, dissolved oxygen; EC, electrical conductivity; NO, nitrate; TDS, total dissolved solids; SO_4_^2−^, sulfate.

For all the evaluated parameters, the treated AMD water displayed a significant difference (p < 0.05) see Table 1. When compared to the untreated 100% AMD water (TW2), the treated AMD water (TW3, TW4, and TW5) had lower concentrations of As, Co, Cu, Cd, Cr, Fe, Mg, Mn, Ni, and Zn (T2). According to^40^ treating AMD with QL eliminated 99% of As, Cd, Co, Cu, Fe, Mn, Ni, and Zn, which is consistent with our findings. Additionally^33^, found that the concentration of As, Cd, and Cr decreased when AMD was treated with 2 g of QL equivalent to 1 L of AMD. Except for Pb, Mg, and Mo, most of the heavy metals in TW4 and TW5 were decreased to levels below those stated in standards when this study’s findings were compared to the stipulated standard^39^. TW3 did not satisfy any recommended standard with As, Cd, Cr, Fe, Ni, and Zn. According to^53^, the differences between the three treatments may be due to the amount of QL used and the addition of fly ash to the QL treatment. High concentrations of metals in irrigation water, according to^9^, contaminate agricultural soils and cause crops grown on these soils to absorb metals. Therefore, it is necessary to maintain irrigation water's heavy metal content within a predetermined threshold.

### Effect of the irrigation water on the physiological parameters, biochemical and yield attributes of potato cultivars

**Figure 3:**
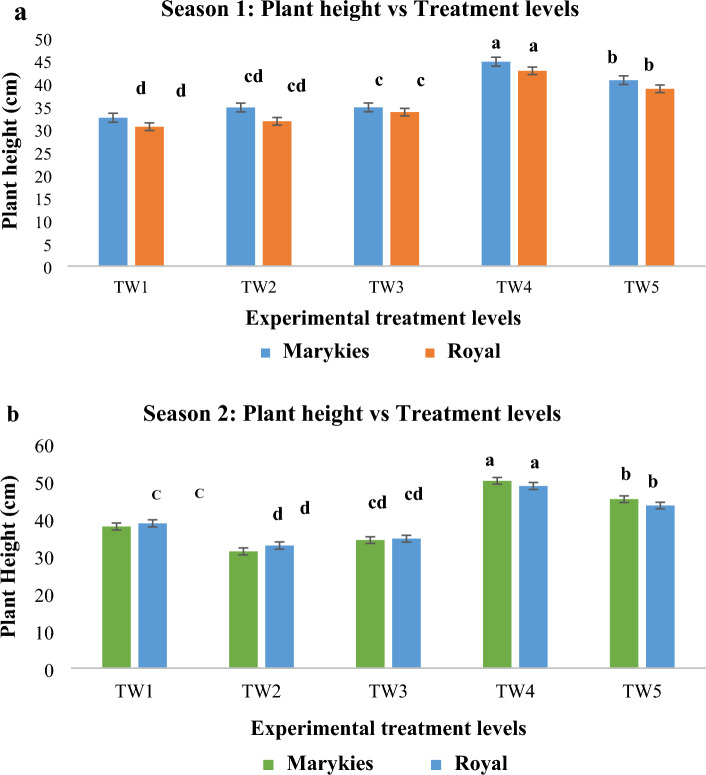
(**a**) and (**b**) Plant height response of two cultivars of potato (Marykies and Royal) irrigated with quicklime and Fly Ash (FA) treated acid mine drainage (Season 1 and 2). Similar letters across the treatments are not significantly different at p ≤ 0.05.
- * Chlorophyll content*. Plants exhibit dynamism that cuts across physiological, metabolic, and molecular responses in their struggle to survive adverse environmental conditions^58^. A study by^59^ stated that chlorophyll concentration, stomatal conductance, and biomass of roots, stems, leaves, and fruits can all be used to determine a plant's physiological growth. Several studies have suggested that factors such as water stress and soil types might affect the chlorophyll concentration of plant leaves^60^. For this study, the chlorophyll content (mg/m^2^) and stomatal conductance (mmol m^2^ s^1^) of two cultivars of potato grown under the irrigation of treated AMD water were measured to ascertain whether their physiological response could promote their survival under (un)treated AMD water stress. The measured physiological parameters showed significant differences (p < 0.05) across the two cultivars. Royal cultivar produced more chlorophyll content than the Marykies (Table 2). Like in these findings^20^, also observed variation in the chlorophyll response when different cultivars of potato were treated with AMD water. This could be linked to crops' physiological responses to stress, which vary depending on the type and level of crops, as well as the type of crops involved^57^. The chlorophyll content (chlorophyll a & b) determined on the leaf of the selected cultivars was significantly affected by long-term irrigation with the different quicklime AMD treatments. For example, the highest chlorophyll content a & b for both cultivars responding to treatments was recorded on TW2 and TW3 as compared to the controls (TW1) and TW4 and TW5 for both seasons (Table 2). The increase in chlorophyll can be due to the possible accumulation of metals (toxic effect) in comparison with the other treatments. A similar trend was observed in season 2 as well. The chlorophyll content varied on days after planting (DAP) with the highest recorded on D5 which is day 56 after planting (56 DAP). There was a trend in that the chlorophyll content decreased as the day of planting increased. Congruent to our study, other studies have also observed variation in the chlorophyll content with DAP as impacted the AMD treatment^20,61^. For this study, the implication is that long exposure of the plants to QL treated AMD could impair the physiological processes of the crops.

- *Plant height*. The effects of irrigation water were reported in Fig. 3a and b to show the response of the two cultivars to the different treatments of AMD water. A significant difference (p < 0.05) was observed across the treatments for both cultivars. In addition, the progress growth of the crops was recorded as shown in Fig. 3a and b. In general, the Marykies cultivar responded better than the Royal across the treatments. This could be attributed to differences in the physiological response of the two cultivars as impacted by their molecular properties under AMD environment^54^. The study conducted by^55^ also indicated that different plants respond differently in the synthesis of proteins that could play a crucial role in their adaptation and survival under AMD conditions. The differential protein abundance in the Marykies or the plant–microbe interactions could have played a crucial role in their better adaptation under the treated AMD condition. However, there is a need for further studies to validate this assumption. Among the treatment levels, TW4 and TW5 enhanced the plant height of the two cultivars better regarding the AMD sample (TW2) and control (TW1). This may be due to lime and fly ash's beneficial benefits in reducing soil acidity, as they are well-known for their strong acid-neutralizing capabilities, which can effectively eliminate existing acid, increase biological activity, and minimize heavy metal toxicity^52,56^. For instance, a study conducted by^57^, applying lime to acid soil increased barley height, fresh biomass, dry biomass, grain yields, harvest index, and P-uptake. Furthermore, the use of lime enhanced maize growth and yield, owing to the reduction in Al toxicity. For this study, TW2 and TW3 exhibited low plant height as compared to TW1, TW4 and TW5. Similar results were reported by^24^ who observed negative effects on the growth and grain yield of winter wheat irrigated with acid mine wastewater.

**Table 2:** Effects of AMD water treated with quicklime on Marykies and Royal cultivars chlorophyll content (Season 1 and 2).

Chlorophyll content for Marykies and Royal potato cultivars					
Cultivars	Season 1		Season 2		
Marykies	16.15 ± 0.44b	27.90 ± 0.57b	26.90 ± 0.57b	33.30 ± 1.00b	
Royal	18.91 ± 0.53a	30.39 ± 0.64a	29.39 ± 0.64a	36.90 ± 1.10a	

Treatments	Season 1			Season 2	
	Treatments vs cultivar chlorophyll content			Treatments vs. cultivar chlorophyll content	
	Trts. vs Cultv	Chlorophyll a	Chlorophyll b	Chlorophyll a	Chlorophyll b
Tap water 0% (TW1)	T1 _Marykies_	16.95 ± 1.21d	19.25 ± 1.46d	17.94 ± 1.28c	19.25 ± 1.57c
	T1 _Royal_	18.09 ± 1.73d	21.49 ± 1.93d	20.09 ± 1.84c	22.49 ± 2.71c
AMD 100% (TW2)	T2 _Marykies_	21.53 ± 2.26a	24.96 ± 3.56a	23.09 ± 2.83a	26.60 ± 3.42a
	T2 _Royal_	24.74 ± 3.52a	27.60 ± 3.87a	25.30 ± 3.04a	28.97 ± 4.64a
1 g + AMD (TW3)	T3 _Marykies_	19.93 ± 1.38b	23.74 ± 2.89b	21.50 ± 2.14b	24.97 ± 3.00b
	T3 _Royal_	23.73 ± 2.86b	25.58 ± 3.05b	23.25 ± 2.86b	26.02 ± 3.21b
2 g + AMD (TW4)	T4 _Marykies_	15.32 ± 0.99e	18.05 ± 1.40e	15.32 ± 0.99e	17.05 ± 1.46e
	T4 _Royal_	17.27 ± 1.28e	19.96 ± 1.61e	18.27 ± 1.36e	20.95 ± 1.69e
2 g + AMD + 75% (TW5)	T5 _Marykies_	16.95 ± 1.13c	19.64 ± 1.58c	16.96 ± 1.13d	18.64 ± 1.46d
	T5 _Royal_	19.84 ± 1.59c	21.20 ± 1.66c	19.63 ± 1.50d	21.20 ± 1.82d

Days after planting (DAP)	Cultivars	Season 1		Season 2	
		Days after planting vs cultivar chlorophyll content		Days after planting vs cultivar chlorophyll content	
		Chlorophyll a	Chlorophyll b	Chlorophyll a	Chlorophyll b
D1	Marykies	12.84 ± 0.28f	15.62 ± 0.25f	13.52 ± 0.32f	16.08 ± 0.32f
D1	Royal	15.43 ± 0.36f	18.47 ± 039f	16.92 ± 0.29f	19.92 ± 0.40f
D2	Marykies	16.53 ± 0.34e	19.36 ± 0.36e	16.55 ± 0.30e	19.18 ± 0.37e
D2	Royal	17.96 ± 0.41e	21.04 ± 0.43e	19.04 ± 0.36e	22.08 ± 0.59e
D3	Marykies	20.13 ± 0.53c	23.23 ± 0.68c	20.68 ± 0.79c	23.86 ± 0.82c
D3	Royal	24.35 ± 0.72c	27.67 ± 0.89c	25.50 ± 1.03c	29.02 ± 1.26c
D4	Marykies	23.00 ± 0.56b	26.63 ± 0.73b	23.68 ± 0.80b	27.49 ± 1.17b
D4	Royal	27.51 ± 0.76b	30.62 ± 0.89b	28.56 ± 1.06b	31.59 ± 0.95b
D5	Marykies	26.41 ± 1.36a	32.05 ± 1.93a	31.21 ± 1.73a	34.97 ± 1.81a
D5	Royal	33.47 ± 0.86a	36.58 ± 0.98a	34.47 ± 1.32a	37.56 ± 1.93a
D6	Marykies	19.14 ± 0.45d	22.32 ± 0.51d	19.87 ± 0.63d	23.27 ± 0.74d
D6	Royal	21.38 ± 0.60d	24.40 ± 0.62d	22.58 ± 0.78d	25.67 ± 0.81d
D7	Marykies	13.14 ± 0.16g	11.12 ± 0.12g	13.75 ± 0.16g	12.13 ± 0.14g
D7	Royal	13.33 ± 0.19g	11.68 ± 0.14g	13.97 ± 0.15g	12.23 ± 0.15g
D8	Marykies	9.22 ± 0.03h	7.20 ± 0.02h	9.86 ± 0.06h	7.78 ± 0.04h
D8	Royal	10.06 ± 0.08h	8.03 ± 0.03h	10.68 ± 0.11h	8.55 ± 0.04h
D9	Marykies	4.94 ± 0.02i	3.48 ± 0.02i	5.12 ± 0.03i	3.81 ± 0.01i
D9	Royal	6.75 ± 0.04i	4.94 ± 0.03i	7.50 ± 0.04i	5.66 ± 0.04i
F-statistics Cultivar		342.51 s	428.50 s	12,325 s	19,903 s
F-statistics Treatments × Cultivar		1.6 s	2.19 s	41 s	63 s
F-statistics Cultivar × DAP		18.40 s	22.97 s	440 s	823 s
F-statistics Cultivar × DAP × Treatments		1.38 s	1.4 s	86 s	184 s

Mean ± SE in the same column with dissimilar letters are significantly different at p < 0.05.
- *Stomatal conductance*. Stomatal conductance is referred to as a measure of the degree of physical resistance to gas movement between air and leaf interior^62^. Such an exchange supports the exchange in CO_2_ intake and water loss (transpiration) through the stomatal aperture. Stomatal adjustments help to maintain plant water status under varying soil moisture and atmospheric conditions. Acid mine drainage is known to constitute an environmental stress to plants, and this culminates into diverse physiological and morphological responses by plants that include reduction in transpiration rate and stomatal conductance^63,64^. The two measured physiological parameters showed significant differences (p < 0.05) across the two cultivars. Royal cultivar produced greater stomatal conductance than the Marykies (Table 3). Investigation from^20^ also observed variation in stomatal conductance between Fiana and Lady Rosetta cultivars that were treated with AMD water. In concurrence with the findings of this study, in other research studies conducted under extreme environmental stress, plants exhibit a plethora of physiological adjustment which reduces stomatal conductance and chlorophyll content as a mechanism to aid their survival^65–68^. The treatment of AMD with QL was promising as the treatments were able to improve the stomatal conductance of the two cultivars. Similarly, there were significant differences (p < 0.05) in the stomatal conductance on DAP across the different days of measurement with D5 also showing the highest stomatal conductance (Table 3). Stomatal conductance continued to decrease as the plant grew older.

**Table 3:** Effects of acid mine drainage (AMD) water treated with quicklime on the stomatal conductance of Marykies and Royal (Season 1 & 2).

Stomatal conductance for Marykies and Royal potato cultivars					
Cultivar	Season 1		Season 2		
	Abaxial	Adaxial	Abaxial	Adaxial	
Marykies	84.08 ± 2.30b	92.65 ± 2.40b	87.15 ± 3.31b	92.55 ± 3.46b	
Royal	102.80 ± 1.85a	111.99 ± 1.85a	106.68 ± 2.74a	116.14 ± 2.81a	

Treatments	Season 1			Season 2	
	Treatments vs cultivar stomatal conductance			Treatments vs cultivar stomatal conductance	
		Abaxial	Adaxial	Abaxial	Adaxial
Tap water 0% (TW1)	T1 _Marykies_	85.35 ± 4.55c	94.45 ± 4.75c	89.12 ± 4.79c	98.87 ± 5.11c
	T1 _Royal_	105.19 ± 3.44c	114.39 ± 3.87c	108.88 ± 5.16c	112.51 ± 5.01c
AMD 100% (TW2)	T2 _Marykies_	120.49 ± 6.42a	126.12 ± 7.02a	107.76 ± 7.96a	118.06 ± 8.04a
	T2 _Royal_	128.38 ± 7.59a	134.16 ± 8.25a	119.77 ± 8.11a	125.48 ± 8.89a
1 g + AMD (TW3)	T3 _Marykies_	103.39 ± 5.34b	113.65 ± 5.83b	100.92 ± 5.06b	108.84 ± 5.10b
	T3 _Royal_	110.30 ± 5.56b	121.96 ± 6.53b	113.40 ± 5.81b	120.01 ± 8.38b
2 g + AMD (TW4)	T4 _Marykies_	83.33 ± 4.88e	91.60 ± 4.09e	86.96 ± 4.44e	94.48 ± 5.31e
	T4 _Royal_	101.00 ± 3.74e	109.74 ± 3.91e	93.60 ± 5.02e	101.96 ± 5.40e
2 g + AMD + 75% (TW5)	T5 _Marykies_	82.31 ± 4.44d	91.29 ± 4.89d	87.16 ± 4.62d	96.65 ± 4.88d
	T5 _Royal_	102.30 ± 3.73d	110.96 ± 3.87d	95.31 ± 4.38d	99.40 ± 5.18d

Days after planting (DAP)	Cultivars	Season 1		Season 2	
		DAP vs Cultivar stomatal conductance		DAP vs Cultivar stomatal conductance	
		Abaxial	Adaxial	Abaxial	Adaxial
D1	Marykies	84.53 ± 0.65g	93.96 ± 0.69g	87.64 ± 0.96g	95.35 ± 0.84g
D1	Royal	89.12 ± 0.71g	97.99 ± 0.98g	92.70 ± 0.67g	101.53 ± 0.98g
D2	Marykies	89.40 ± 0.78f	99.89 ± 1.01f	93.70 ± 0.81f	102.63 ± 1.03f
D2	Royal	96.06 ± 0.75f	105.51 ± 1.13f	98.48 ± 0.95f	108.25 ± 1.25f
D3	Marykies	102.71 ± 1.09e	113.53 ± 1.24e	109.59 ± 1.36e	120.92 ± 1.95e
D3	Royal	103.57 ± 1.10e	112.09 ± 1.19e	106.94 ± 1.33e	115.33 ± 1.86e
D4	Marykies	118.93 ± 1.27b	127.30 ± 1.37b	122.44 ± 1.97b	131.10 ± 2.77b
D4	Royal	122.55 ± 1.26b	131.48 ± 1.64b	128.14 ± 2.21b	138.08 ± 2.93b
D5	Marykies	128.46 ± 1.40a	137.99 ± 1.82a	130.10 ± 2.65a	140.86 ± 3.02a
D5	Royal	133.10 ± 1.69a	143.37 ± 1.95a	138.17 ± 2.97a	150.32 ± 3.71a
D6	Marykies	113.82 ± 1.21c	123.45 ± 1.47c	116.92 ± 1.47c	125.39 ± 2.12c
D6	Royal	141.76 ± 2.01c	150.62 ± 2.87c	146.47 ± 3.58c	153.85 ± 3.96c
D7	Marykies	73.47 ± 0.28d	82.68 ± 0.42d	75.48 ± 0.58d	84.38 ± 0.72d
D7	Royal	116.65 ± 1.49d	124.94 ± 1.59d	120.47 ± 1.68d	129.28 ± 1.83d
D8	Marykies	32.64 ± 0.13h	39.33 ± 0.21h	34.57 ± 0.19h	41.97 ± 0.26h
D8	Royal	82.77 ± 0.36h	92.45 ± 0.59h	84.17 ± 0.76h	93.11 ± 0.65h
D9	Marykies	12.73 ± 0.09i	15.67 ± 0.09i	13.85 ± 0.06i	17.12 ± 0.09i
D9	Royal	39.60 ± 0.09i	43.48 ± 0.06i	41..42 ± 0.28i	49.55 ± 0.84i
F-statistics					
Cultivars		2059.10 s	2255.70 s	72,761 s	114,492 s
Treatment × Cultivar		1.7 s	0.6 s	82 s	32 s
Cultivar × DAP		263.9 s	246.3 s	12,656 s	7820 s
Cultivar × DAP × Treatments		2.8 s	2.6 s	156 s	106 s

Mean ± S.E values followed by similar letters in a column are not significantly different at p ≤ 0.05.
- * Yield parameters and their components. *A two-way ANOVA analysis showed that the quicklime and fly ash treatments of AMD were significant (p < 0.05) across the treatments for the tuber yield, fresh tuber weight, and dry tuber weight for both cultivars (Table 4) with subtle variation between them. The treated AMD water samples (TW3, TW4, and TW5) improved all the yield parameters for the two potato cultivars with T2 showing higher potential in the improvement of the yield (Table 4). Maize (*Zea mays*) and sunflower (*Helianthus annuus*) grown in a heavy metal-enriched AMD environment showed enhanced growth and copper uptake, as reported in the^69^ findings. Additionally, using copper-resistant Pseudomonas strains improved Zn and Pb bioaccumulation as well as plant growth-promoting indole-3-acetic acid (IAA), iron chelating siderophore, and mineral phosphate and metals solubilization capacity^70^. The increased crop yield observed under TW2 could have been because of the presence of growth-promoting bacteria that could have promoted the growth promotion and heavy metal bioaccumulation of the potatoes but might have also enhanced the remediation function through plant microbes interactions^71^. Results on season 2 (Table 4) revealed a slight variation between treatments compared to season 1. Marykies had a higher number of tubers on TW4 and TW5 and this enhancement in yield can be in response to quicklime and fly ash application and other environmental factors in the greenhouse. Several research including^72–74^ reported that fly ash has the potential to improve the yield of wheat (*Triticum aestivum*), rice (*Oryza sativa*), maize (*Zea mays*), mung bean (*Vigna unguiculata*), eggplant (*Solanum melongena*), onion (*Alium cepa*) and chickpea (*Cicer arietinum*) cultivated on different types of soils. Irrigation with AMD water generally causes a shift in the parameters of soils, has the potential to positively alter microbial diversity and plays vital roles in the ecology of the rhizosphere of plants through the maintenance of soil health and therefore increasing the yield of crops^75–77^. The decrease in the yield of crops irrigated with treated AMD water could be a function of the important microbe reduction during the process of treatment^6^. Hence, there is a need to evaluate a system where AMD treatment can protect the important microbial communities while removing the harmful substance that plants can translocate from the soil.

**Table 4:** Effects of treated quicklime AMD water on the Marykies and Royal yield.

	Cultivar	No. of tubers	Fresh tuber weight (g)	Dry tuber weight (g)
Season 1	Marykies	9.07 ± 0.30a	123.15 ± 2.13b	45.93 ± 1.17b
	Royal	6.47 ± 0.41b	236.38 ± 9.80a	79.16 ± 4.68a
Season 2	Marykies	8.27 ± 0.55a	122.19 ± 2.82b	45.56 ± 2.17b
	Royal	6.33 ± 0.34b	287.08 ± 9.91a	91.90 ± 4.80a

Season 1 Treatments				
	Trts. vs Cultv	No. of tubers	Fresh tuber weight (g)	Dry tuber weight
Tap water 0% (TW1)	T1 _Marykies_	9.00 ± 0.57c	120.27 ± 0.17a	42.93 ± 0.06a
	T1 _Royal_	7.00 ± 0.57c	310.25 ± 40.37a	95.10 ± 12.23a
AMD 100% (TW2)	T2 _Marykies_	8.00 ± 0.57e	122.22 ± 0.08b	46.34 ± 1.06b
	T2 _Royal_	7.33 ± 1.20e	257.33 ± 53.76b	83.51 ± 13.33b
1 g + AMD % (TW3)	T3 _Marykies_	9.00 ± 1.00d	137.70 ± 0.89e	53.30 ± 0.57e
	T3 _Royal_	5.00 ± 0.57d	153.79 ± 13.21e	64.88 ± 1.80e
2 g + AMD % (TW4)	T4 _Marykies_	9.67 ± 0.67a	113.90 ± 1.69c	42.20 ± 1.15c
	T4 _Royal_	6.33 ± 1.45a	247.02 ± 48.09c	82.19 ± 12.83c
2 g + AMD % + 75% FA	T5 _Marykies_	9.67 ± 0.33b	121.68 ± 0.31d	45.68 ± 1.86d
	T5 _Royal_	6.67 ± 0.23b	213.50 ± 6.64d	70.11 ± 0.28d
F-statistics treatment				
Cultivars		25.77 s	45.42 s	56.60 s
Treatment		0.66 s	1.88 s	0.78 s
Treatment × Cultivar		1.29 s	2.95 s	2.55 s

Season 2 Treatments				
	Trts. vs Cultv	No. of tubers	Fresh tuber weight	Dry tuber weight
Tap water 0% (TW1)	T1 _Marykies_	7.33 ± 0.67d	122.87 ± 1.74a	46.20 ± 0.22a
	T1 _Royal_	6.00 ± 0.58d	327.07 ± 6.58a	106.26 ± 0.93a
AMD 100% (TW2)	T2 _Marykies_	10.67 ± 0.67a	136.32 ± 1.48b	55.77 ± 1.57b
	T2 _Royal_	8.00 ± 0.58a	300.26 ± 19.96b	103.75 ± 2.9ba
1 g + AMD % (TW3)	T3 _Marykies_	7.33 ± 0.33c	120.29 ± 0.36c	42.67 ± 1.18c
	T3 _Royal_	6.33 ± 0.67c	287.07 ± 17.65c	100.48 ± 3.15c
2 g + AMD % (TW4)	T4 _Marykies_	5.67 ± 0.30e	105.10 ± 0.41d	33.70 ± 0.42d
	T4 _Royal_	6.33 ± 0.38e	260.54 ± 11.57d	78.67 ± 11.02d
2 g + AMD % + 75% FA	T5 _Marykies_	10.00 ± 1.00b	126.35 ± 4.05e	49.47 ± 5.06e
	T5 _Royal_	5.00 ± 0.57b	240.45 ± 11.36e	70.33 ± 11.67e
F-statistics treatment				
Cultivars		25.49 s	623.24 s	175.22 s
Treatment		8.67 s	7.25 s	6.88 s
Treatment × Cultivar		5.94 s	4.98 s	3.97 s

Mean ± S.E. values followed by similar letters in a row are not significantly different at p ≤ 0.05.

### Effect of water quality on potato tubers and irrigated soil

Due to the potential for crops to absorb heavy metals, irrigation of crops with AMD water, whether it is sourced from industrial, municipal, or sewage and whether it has been treated or not, has been documented to be harmful to crops and agricultural soil^9,78^. In this study, quicklime and fly ash were used to water different potato cultivars, and the number of heavy metals in the tubers was measured. The findings revealed that heavy metals were present in the tubers and that their quantities varied significantly (p ≤ 0.05) depending on the treatments Table 5. When compared to the 100% AMD, there was a decrease in the concentration of several heavy metals in the tubers, especially after quicklime and fly ash treatments (TT3, TT4, and TT5) (TT2). This is probably due to the treatment's success in lowering the level of heavy metal in the 100% AMD. The creation of several metabolites that are essential for the signalling, sequestration, and transportation of heavy metals like Fe, Cu, Zn, and Cd is observed to rise because of heavy metal stress, according to several studies^79–83^. The concentration of some of the heavy metals decreased, however not all the lowered heavy metal concentrations fell under the^39^ recommended tolerable limits. The following heavy metals such as Al, Co, Cu, Fe, Mg, Mn, Ni, and Zn met the requirements. In contrast, the levels of As, Cd, Cr, Pb, and Mo were higher than what is considered safe by^39^ criteria. Hence, further studies that could unveil the underlying shift in a metabolite that could be responsible for the crop not sequestrating many of the heavy metals are recommended. Such research may also shed light on the mechanisms underlying the heavy metal hyperaccumulation capacity of certain potato cultivars. Additionally, increased concentrations of heavy metals such As, Cd, Pb, and Zn were found in the soils and vegetables grown in Portugal under irrigation using water from locations near mines^84^. While ^85^ revealed that fresh vegetable samples that had As and Pb concentrations that were higher than allowed by international food standards had contaminated irrigation in areas close to mining zones. For example, in Guangdong province, South China, around the Lechang Pb/Zn mine^86^, high levels of metals such as Cd, Pb, Cu, and Zn were measured in 11 edible vegetables, including *Solanum tuberosum* (potato). The results revealed that local mining activity caused heavy metal contamination, with Cd concentration exceeding the required standards for all vegetables. In another study, paddy fields in a karst region of Guangxi Province, South China, were found to be heavily contaminated with Cd, Zn, Pb, and Cu^87^. In the same Lechang mining area, Guangdong Province, South China, irrigation with mining effluent contaminated a paddy field and rice grain with Cd^88^. According to a study by^89^, Pb and Cd concentrations in rice grain are above China's maximum allowable limits. The study looked at the extent to which heavy metals (Cu, Zn, Pb, and Cd) contaminate soils, vegetables, and rice growing near the Dabaoshan mine in South China. In Potosi (Bolivia), it was shown that potato tubers irrigated with streams affected by mining had higher Cd concentrations than those irrigated with spring water^18^. Similarly, in this study, higher than required levels of As, Cd, Pb, and Zn were found in the heavy metal content of potato tubers cultivated in acid mine water released from mining enterprises in Potosi^19^.

**Table 5:** Mean ± standard deviation of the potato tubers (Marykies and Royal) heavy metal concentration irrigated with treated AMD in comparison with the permissible limits of World Health Organization standards^39^.

Metals	Heavy metal mean concentration for potato tubers (mg/kg)										
	Marykies tuber					Royal tuber					WHO
	TT1	TT2	TT3	TT4	TT5	TT1	TT2	TT3	TT4	TT5	Limits
As	0.04 ± 0.00e	33.39 ± 0.31a	12.79 ± 0.06b	5.04 ± 0.06d	6.54 ± 0.24c	0.07 ± 0.03e	33.81 ± 0.29a	13.04 ± 0.01b	5.59 ± 0.00d	6.89 ± 0.27c	0.1–0.2
Cd	0.21 ± 0.00e	6.80 ± 0.04a	3.08 ± 0.02b	1.96 ± 0.01d	2.05 ± 0.01c	0.36 ± 0.00e	7.20 ± 0.08a	3.64 ± 0.04b	2.13 ± 0.01d	2.14 ± 0.01c	0.02–0.2
Co	0.02 ± 0.00e	5.48 ± 0.15a	0.17 ± 0.00b	0.07 ± 0.00d	0.10 ± 0.00c	0.03 ± 0.00e	5.93 ± 0.01a	0.23 ± 0.00b	0.08 ± 0.00d	0.10 ± 0.00c	0.05–0.1
Cr	0.70 ± 0.02e	4.96 ± 0.01a	3.47 ± 0.03b	2.83 ± 0.07c	2.57 ± 0.01d	0.80 ± 0.01e	5.03 ± 0.01a	3.59 ± 0.04b	3.01 ± 0.01c	2.95 ± 0.01d	1.3
Cu	2.90 ± 0.05e	47.83 ± 0.08a	11.56 ± 0.15b	5.02 ± 0.06c	3.12 ± 0.02d	3.40 ± 0.08e	50.25 ± 0.46a	12.19 ± 0.09b	5.54 ± 0.05c	3.95 ± 0.01d	10–60
Fe	2.81 ± 0.01e	46.55 ± 0.12a	14.76 ± 0.23b	4.54 ± 0.01c	3.16 ± 0.00d	3.02 ± 0.05e	49.18 ± 0.07a	16.52 ± 0.09b	4.97 ± 0.01c	3.72 ± 0.05d	425
Mg	36.03 ± 0.30b	62.50 ± 0.53a	17.70 ± 0.09e	28.03 ± 0.18c	26.12 ± 0.11d	37.38 ± 0.29b	65.05 ± 0.19a	19.76 ± 0.17e	30.72 ± 0.13c	29.08 ± 0.12d	–
Mn	7.80 ± 0.12d	46.11 ± 0.09a	14.80 ± 0.17b	8.04 ± 0.04c	6.26 ± 0.06e	8.28 ± 0.04d	49.40 ± 0.27a	15.09 ± 0.06b	8.41 ± 0.05c	6.89 ± 0.01e	500
Mo	0.35 ± 0.03e	13.65 ± 0.00a	2.37 ± 0.09b	1.61 ± 0.03d	1.91 ± 0.05c	0.44 ± 0.02e	14.07 ± 0.02a	2.84 ± 0.03b	1.99 ± 0.05c	1.60 ± 0.02d	100
Ni	0.28 ± 0.00e	13.03 ± 0.01a	2.31 ± 0.01b	1.65 ± 0.00c	0.75 ± 0.00d	0.30 ± 0.00e	13.51 ± 0.09a	2.91 ± 0.01b	1.93 ± 0.01c	0.95 ± 0.14d	10
Pb	0.07 ± 0.00e	45.92 ± 0.06a	17.82 ± 0.01b	4.95 ± 0.03c	3.40 ± 0.03d	0.09 ± 0.00e	46.07 ± 0.01a	17.96 ± 0.01b	5.08 ± 0.01c	3.50 ± 0.01d	0.3–2.0
Zn	7.05 ± 0.04e	164.82 ± 0.77a	20.63 ± 0.01b	9.37 ± 0.10c	8.25 ± 0.05d	7.53 ± 0.02e	170.85 ± 0.12a	21.08 ± 0.01b	10.30 ± 0.02c	8.89 ± 0.07d	23

The study also evaluated the concentration of selected heavy metals in the soils irrigated with quicklime and fly ash-treated AMD. An analysis was done to delineate the impact of heavy metal contamination of the soil on the crops (Table 6). Studies have shown that when plants are raised in soils that are contaminated with heavy metals, they absorb and accumulate these in their edible parts of plants and these could be beyond the permissible limits, which could be harmful to humans it consumed^90^. The present results showed significant differences (p < 0.05) across the treatments for both cultivars. The soil irrigated with treated AMD (ST3, ST4, and ST5) showed variation in the concentrations of heavy metals and were within the permissible limit of the WHO except for As, Cd and Cr. As expected, the soil that was irrigated with untreated AMD water (ST2) showed a higher concentration of heavy metals in most of the measured metals that were not within the stipulated standard. This is due to the transfer of heavy metals from the untreated AMD and the inability of the crops to sequester such high concentration^91^. In agreement with these findings, some studies have also reported an increase in the concentration of heavy metals in soil polluted with heavy metal-laden waste^92^. A study by^93^ investigated the degree of contamination of heavy metals in paddy soil irrigated with acid mine drainage and showed that Cu, Zn, and Cd in topsoil exceeded the maximum permissible concentrations for Chinese agricultural soil. Another study^91^ observed similar findings on paddy fields that had been extensively polluted by Cu, Zn, and Cd due to long-term irrigation with nearby stream water contaminated by acid mine wastes. In their findings^94^, reported higher concentrations of heavy metals and soil salinity during the experimental period for plots irrigated with mine wastewater, when compared to plots irrigated with fresh water. Overall, a significant number of studies on heavy metals in plants have been conducted in Chinese paddy fields, which is likely due to a large amount of mining in that region of the world, which has resulted in AMD accumulation^,^^23,95^.

**Table 6:** Mean ± standard deviation of the soil heavy metal concentration irrigated with treated AMD and tapwater (control) in comparison with the acceptable level of World Health Organization (WHO) standards and Department of Environmental Affairs (DEA: standards).

Metals	Heavy metal mean concentration for the soil (mg/kg)											
	Soil for Marykies cultivar					Soil for Royal cultivar					mg/kg	
	ST1	ST2	ST3	ST4	ST5	ST1	ST2	ST3	ST4	ST5	WHO	DEA
As	7.88 ± 0.21	45.46 ± 0.84	38.74 ± 0.92	22.85 ± 0.49	24.93 ± 1.01	9.97 ± 0.01e	46.94 ± 0.28a	39.57 ± 0.26b	23.51 ± 0.24c	25.97 ± 1.15d	20	5.5
Cd	0.51 ± 0.00e	22.99 ± 0.43	10.26 ± 0.40	3.52 ± 0.04	5.35 ± 0.01	0.60 ± 0.00e	23.92 ± 0.38a	10.86 ± 0.64b	3.66 ± 0.01d	5.38 ± 0.02c	3	–
Co	30.51 ± 0.58	60.57 ± 1.59	47.92 ± 0.94	40.20 ± 0.05	36.92 ± 0.20	31.41 ± 0.14e	62.20 ± 0.57a	50.05 ± 0.22b	42.72 ± 0.06c	38.36 ± 1.07d	50	300
Cr	1.30 ± 0.01e	141.22 ± 0.2.31	30.30 ± 0.04	11.94 ± 0.09	17.88 ± 0.04	1.43 ± 0.03e	143.61 ± 1.05a	30.39 ± 0.03b	12.14 ± 0.03d	18.15 ± 0.01c	–	6.5
Cu	3.83 ± 0.01d	19.74 ± 0.77	9.30 ± 0.03	4.28 ± 0.11	2.13 ± 0.01e	3.99 ± 0.05d	20.50 ± 0.57a	9.57 ± 0.18b	4.84 ± 0.04c	2.19 ± 0.05e	100	16
Fe	51.03 ± 0.01e	1747.50 ± 8.42a	705.45 ± 1.06b	177.99 ± 0.57c	79.02 ± 0.32d	51.08 ± 0.01e	1743.60 ± 6.16a	701.13 ± 1.87b	174.64 ± 0.18c	78.30 ± 0.31d	5000	–
Mg	19.76 ± 0.17d	213.26 ± 0.77a	66.81 ± 0.71b	26.12 ± 0.11c	10.79 ± 0.02e	18.93 ± 0.02d	211.18 ± 0.85a	65.99 ± 0.67b	27.90 ± 0.03c	10.68 ± 0.01e	–	–
Mn	34.80 ± 0.09c	42.56 ± 0.37a	36.32 ± 0.16b	13.34 ± 0.57d	7.88 ± 0.09e	35.03 ± 0.74c	44.46 ± 0.11a	35.64 ± 0.10b	13.92 ± 0.05d	8.08 ± 0.05e	740	2000
Mo	ND	ND	2.35 ± 0.17c	6.85 ± 0.11b	10.78 ± 0.18a	ND	ND	2.99 ± 0.05c	7.53 ± 0.05b	10.26 ± 0.24a	5	–
Ni	31.87 ± 0.13d	63.05 ± 1.43a	42.80 ± 0.53b	32.62 ± 0.11c	30.31 ± 0.26e	31.83 ± 0.25d	62.63 ± 0.09a	45.13 ± 0.41b	33.74 ± 0.20c	31.01 ± 0.12e	50	91
Pb	2.65 ± 0.04e	32.55 ± 0.04b	38.43 ± 0.02a	26.06 ± 0.27c	22.98 ± 0.13d	2.69 ± 0.04e	33.13 ± 0.02b	40.01 ± 0.18a	26.51 ± 0.07c	23.06 ± 0.15d	100	20
Zn	5.09 ± 0.00c	345.89 ± 4.34a	3.41 ± 0.05e	5.61 ± 0.07b	4.44 ± 0.32d	5.12 ± 0.00c	352.30 ± 3.46a	3.46 ± 0.0e	5.81 ± 0.05b	4.79 ± 0.13d	300	240

### Soil-to-plant transfer factor

**Figure 4:**
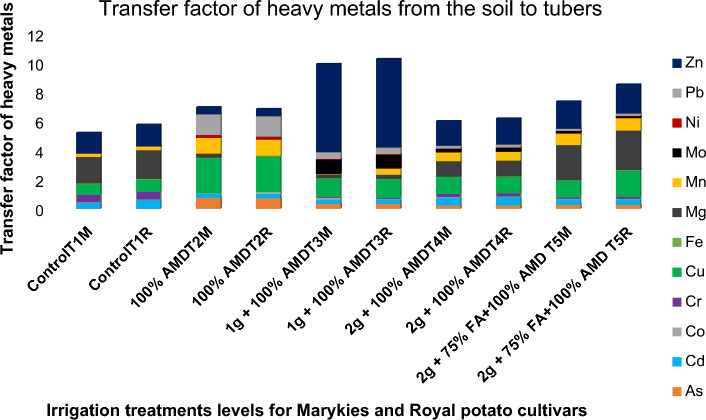
Transfer factor of heavy metals from soil to potato tubers irrigated with quicklime (un)treated AMD. Different letters indicate significant differences (Duncan’s test, p < 0.05), among metals.

PTF provides a useful indication of the metal availability from soil to plants. The PTF values for Cd, Cu, Fe, Ni, Mn, Pb and Zn ranged from 0.01 to 5.17, 0.25 to 1.80, 0.00 to 0.01, 0.00 to 2.90, 0.04 to 0.10, 0.16 to 2.90 and 0.14 to 0.52, respectively. The mean value of PTF for each heavy metal is shown in Fig. 4. The higher the value of the transfer factor, the more elements would be accumulated by plants. The trend of transfer factor and thus the availability of heavy metals for potatoes was in the order of Sr > Zn > Cu > Mg > Mn > Pb > Cd > As > Mo > Cr > Ni > Fe and Co. The availability of heavy metals in potato tubers was significantly (P < 0.05) different among heavy metals and the accumulation of Cd in potatoes was highest. These results agree with previous investigations. The findings^96^ reported that the transfer factor for Cd and Cu was higher than other metals in vegetables. The other findings^97,98^ reported that the accumulation of Cd and Zn was higher than that of Ni in rice. There was a significant correlation between total Cd concentrations in soil and two potato cultivars (Fig. 4). There was no significant correlation between other metal concentrations in soils.

## Conclusion

This research aimed to evaluate the possible use of quicklime to treat acid mine water for crops and assess the effects on soil properties and potato cultivars Marykies and Royal physiological, biochemical, and yield parameters when subjected to quicklime treated acid mine drainage irrigation under greenhouse conditions. It was found that the quicklime-treated mine water had increased pH levels indicating normal alkalinity which is equivalent to the permissible limits. The observed pH levels and metal removal capacity during the experimental period indicate the potential long-term effectiveness of quicklime in treating AMD. The results showed that soil irrigated with treated AMD water exhibited a noticeable decrease in pH and EC, as well as an increase in sulphate content when compared to the treated AMD water. This might be explained by how plants and bacteria interact, which has the potential to change the soil's ecology and make it more conducive to crop growth. The presence of sulphate-oxidizing bacteria in the AMD is linked to the rise in sulphates in the environment that is polluted by AMD. Since water is a scarce resource in South Africa, these findings make it possible to consider the possibility of using treated AMD in agriculture, without negative consequences to plants and by extension, to human life. Since AMD is available abundantly and quicklime is also cheap to obtain, this study presents a great opportunity to ameliorate AMD water for food security. Soil-to-plant transfer factor revealed that there were high concentrations of total heavy metals in soil and potatoes and that the PTFs were higher for Zn, Cu, Mg, Pb and Mn than other metals. Thus, the transfer of Pb from soils to potatoes may exhibit potential health risks for people who regularly consume heavy metals contaminated potatoes. However, to avoid the eventual risks, the use of AMD must be regularly monitored, and the reuse standards should be developed and strictly observed.

## Data Availability

The datasets generated and/or analysed during the current study are not publicly available due to university policy but are available from the corresponding author on reasonable request.
